# Comparative Study of Genome Divergence in Salmonids with Various Rates of Genetic Isolation

**DOI:** 10.1155/2013/629543

**Published:** 2013-07-24

**Authors:** Elena A. Shubina, Mikhail A. Nikitin, Ekaterina V. Ponomareva, Denis V. Goryunov, Oleg F. Gritsenko

**Affiliations:** ^1^Belozersky Institute for Physico-Chemical Biology of Lomonosov Moscow State University, Leninskie gory, 1, Moscow 119991, Russia; ^2^Biological Department of Lomonosov Moscow State University, Leninskie Gory 1, Moscow 119991, Russia; ^3^Russian Federative Research Institute of Fisheries and Oceanology, 17A V. Krasnoselskaya Street, Moscow 107140, Russia

## Abstract

The aim of the study is a comparative investigation of changes that certain genome parts undergo during speciation. The research was focused on divergence of coding and noncoding sequences in different groups of salmonid fishes of the Salmonidae (*Salmo, Parasalmo, Oncorhynchus*, and *Salvelinus* genera) and the Coregonidae families under different levels of reproductive isolation. Two basic approaches were used: (1) PCR-RAPD with a 20–22 nt primer design with subsequent cloning and sequencing of the products and (2) a modified endonuclease restriction analysis. The restriction fragments were shown with sequencing to represent satellite DNA. Effects of speciation are found in repetitive sequences. The revelation of expressed sequences in the majority of the employed anonymous loci allows for assuming the adaptive selection during allopatric speciation in isolated char forms.

## 1. Introduction

In view of the biological concept of Mayr [1] the process of speciation in the organisms with the sexual reproduction involves accumulation of differences sufficient to set the barrier of partial or complete incompatibility. According to Dobzhansky [2] it implies for the process of unlimited genetic recombinations within the species and the lack of gene flow between the species. Meanwhile, as repeatedly noted by many researches, for example, Mallet, Garside and Christie, Svardson, Wolf et al., Gross et al., and Scribner et al. ([3–7], review [8]), hybridization between species is known to occur both in the wild and under artificial conditions, and the hybrid forms exist along with the parental species. The fate of such interspecific hybrids sporadically occurring in the wild and their contribution in the genetic structure of populations are still under question, as Coyne and Orr and Hudson et al. [9, 10] showed. 

Repetitive DNA sequences are convenient for the studies of the genome evolution [11–13]. According to Ohno [14], this fraction originates in the process of gene duplications and has a potential for large-scale rearrangements, because they are not subjected to the pressing of the natural selection.

From the directly obtained experimental data, phylogenetic reconstructions for the lower taxa on the basis of the repetitive DNA sequences yield better results than the other nuclear sequences for both animals and plants as Chase et al., Thompson et al., and Warburton and Willard [15–17] wrote. As mentioned by Ohta, [18], the factors of intragenomic homogenization counteract intragenomic differentiation of the fraction of repeats. These sequences become peculiar specific markers. The process of concerted evolution has been previously shown by Zimmer et al., Jeffreys et al., Gray et al., and Elder and Turner [19–22] to involve highly repetitive DNA sequences (satellite and satellite-like). 

A well solution method of comparative studies of genomic eukaryotic DNA according to distribution of the sites of digestion by restriction endonucleases was proposed by Fedorov et al. [23]. The method, called taxonomic fingerprinting or taxonoprint, is a modification of the approach initially developed for the analysis of the mitochondrial DNA [24]. Investigation of about 50 animal species of various taxa [25] has shown species specificity of the band patterns along with the absence of individual, sexual, and interpopulation polymorphism. Dominating contribution of high-copy, relatively long tandem repetitive sequences in “taxonoprints” was revealed by Roudykh et al. [26]. This method seems to us to be sufficiently appropriate for studying the molecular aspects of speciation. 

We have shown previously that these general principles of the concert evolution were fully pronounced in the evolution of repetitive sequences of the salmonids of *Salmo*, *Parasalmo,* and *Oncorhynchus* genera—Mednikov et al. [27]. “Homing” common to salmonids resulted in reliable reproductive isolation with the subsequent divergence of the populations in accordance with the morphological and molecular characters. Situation with the repetitive DNA sequences in the organisms with the less strict genetic isolation seemed to be worth being analyzed. Whitefishes of Coregonidae family are one of the largest groups with interspecific hybridization; according to many experts, application of Mayr's biological species concept [1] to these fishes is limited (e.g., see discussion of the problem [28–30]).

The study was aimed at the comparative investigation of alteration of some genome fractions under differentiation of salmonid species and forms belonging to various groups and demonstrating various rates of reproductive isolation. Whitefishes of Coregonidae family, true salmons of the *Salmo*, *Parasalmo* (*Oncorhynchus*, after Smith and Stearley [31]) and *Oncorhynchus* genera, and a number of forms and species of *Salvelinus* genus were studied. The rates of isolation in whitefish and true salmon have polar characteristics due to extensive hybridization in some species and strict homing in the other. Geographic isolates and insular populations of anadromous chars occupy intermediate position. 

Two methods of multilocus DNA analysis were applied in the phylogenetic and taxonomic studies of the Coregonidae and Salmonidae families. The methods were based both on the comparison of the extensive repetitive sequence [26] and collation of the anonymous PCR products (RAPD amplification in modification of Welsh and McClelland [32] and Williams [33]) and their subsequent sequencing.

## 2. Materials and Methods

**Figure 1:**
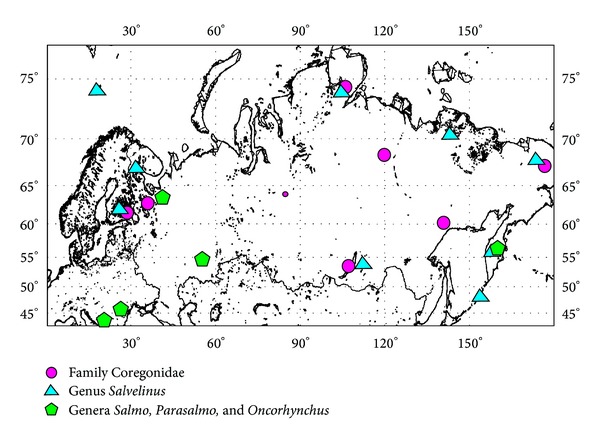
Sampling localities in Russia, Armenia, Kazakhstan, Finland, and Norway.

Most of the salmon tissue samples were collected by the specialists of the Department of Ichthyology, Moscow State University, in 1984–2004 and Russian Federal Research Institute of Fisheries and Oceanography in 2000–2004. Arctic chars from the lakes of Finland were collected by M. Kaukkoranta. Whitefish from Lake Como (Canada) were kindly provided by Yu. S. Reshetnikov. Collecting sites are mapped on Figure 1.

In the early experiments, DNA samples of salmons and whitefish were extracted from gonads at III-IV stages of maturity preserved in alcohol with the method of phenol-chloroform extraction [34]. In some cases, additional purification with CsCl in the presence of ethidium bromide [35] and additional precipitation with cetyltrimethylammonium bromide [36] were performed. The length of DNA was checked by electrophoresis in 0.6% agarose gel. Concentrations of the obtained DNA preparations were estimated on SP-800 spectrophotometer (UK) and adjusted. 

At a later stage, either Silica method [37] or traditional procedure with the use of proteinase K and organic solvents of Sambrook et al. [38] was applied. Prior to amplification, all samples were additionally purified with PEG-8000. DNA pellets were dissolved in TE buffer and stored frozen. 

For restriction analysis DNA aliquots were digested by *Msp*I, *Taq*I, *Csp6*I (*Rsa*I), *Tru9*I (*Ms*eI), *Hin6*I (*Hha*I), and *Mbo*I tetranucleotide restriction endonucleases and two isoschizomers sensitive to the presence of methylated bases (*Bsp143*I and *Sau3A*I), as well as *Cfr13*I and *Bcn*I degenerate pentanucleotide restriction endonucleases (*Fermentas*, Lithuania; *Sibenzym*, Russia). Reactions were performed overnight under conditions corresponding to the manufacturer's recommendations. The fragments of hydrolysis were labeled at cohesive 3′ ends using Klenov's fragment of DNA-polymerase I *E. coli* and using [*a*-^32^P] dNTPs (Institute for Physics and Power Engendering, Obninsk, Russia). Prior to electrophoresis, minor labeled oligonucleotides were removed from hydrolysate by gel filtration through Sefadex G-50 (medium) (Sigma, USA) during centrifugation according to Maniatis et al. [39]. This procedure improved resolution of radioautographs. Electrophoresis in nondenatured 10% polyacrylamide gel (20 × 40 cm) was performed manually according to the method described in Fedorov et al. [23]. pBR322 DNA-*Msp*I digest was used as a marker of molecular weight.

For RAPD-PCR experiments 19- or 20-mer oligonucleotides in various combinations were used as the primers (Tables 1 and 2).

Amplification reaction with two arbitrary primers was performed in 25 *μ*L of 0.01 M tris-HCl PH 8.3 buffer containing 0.05 M NaCl, the mixture of four dNTP (0.2 mM each), MgCl_2_ (5 mM), two arbitrary primers in various combinations (5 *μ*m each), DNA-polymerase *Thermus aquaticus* (2.0 units per sample) (*Dialat*, Russia), and appr. 20 ng of DNA. PCR conditions: 94°C, 2.0 min, (88°C—1 min, 92°—1 min)  ×  1; (94°C—45 sec, 50°C—30 sec, 72°C—30 sec)  ×  3; (94°C—45 sec, 60°C—30 sec, 72°C—30 sec)  ×  35, 72°C—10 min. No DNA was added to the check sample. Compounding ingredients were prepared and mixed in accordance with published recommendations. Electrophoretic fractionation of PCR products was performed in 2% agarose gel (1.5% low melted, Sigma,  +0.5% type II, Sigma), dyed with ethidium bromide, and photographed in UV light. Under the described conditions the reactions were stable and replicable. Photographs of gels containing DNA PCR-RAPD electrophoretic patterns were taken in UV light in Kodak EDAS 290 Electrophoresis Documentation and Analysis System. Adobe Photoshop 7.0 and Gel-Quant (Free Trial) software was used for further editing. Subsequent compilation of gels and construction of the binary matrix of the characters was performed manually. The matrix was compiled according to the “presence or absence of the fragment” principle; only stable major bands were considered. 

Cluster analysis with constructing dendrograms was performed by the distant Neighbor Joining (NJ) approach of Saitou and Nei [40] and UPGMA. Pairwise genetic distances between patterns were calculated according to the method of Nei and Li [41] and Nei [42]. Distant trees were constructed using the TREECON 1.3b program of Van De Peer and De Wachter [43]. Node stability was tested by bootstrap analysis according to Felsenstein [44], and not samples but characters were estimated in both cases. 

DNA fragments were extracted following electrophoretic separation of the PCR products in the agarose gel in columns with GFX PCR: DNA and Gel Band Purification kit (Amersham Biosciences Inc., USA) according to the manufacturer's recommendation. For sequencing of heterogeneous matrices and fragments amplifying from a single primer, corresponding products were cloned in *E. coli* with InsTAclone PCR Cloning Kit (Fermentas, Lithuania) following their extraction and freeing from agarose. The kit included the so-called T-vector, that is, pTZ57R/T plasmid with the extended “sticky” T-end. The colonies of white color containing inserts were grown after screening in liquid medium. In total 160 clones were examined. The difference between lengths ranged from 30 to 50 bp in different cases.

DNA sequencing was performed using ABI PRISM BigDye Terminator v. 3.1 kit with subsequent analysis of the reaction products on ABI PRISM 3100-Avant Genetic Analyzer (Life Technologies/Applied Biosystems, USA). CHROMAS, DNA, and BioEdit programs were used for interpretation of chromatograms. Homologous sequences were searched for in GenBank with the use of dbEST bases from NCBI resources; BLAST software was used for the search.

## 3. Results and Discussion

### 3.1. Restriction Endonuclease Analysis

Restriction analysis of salmonid repetitive DNA has revealed some peculiarities of electrophoretic patterns in whitefishes. The first of them refers to the total number of bands of low intensity, which is extremely high. The matrices are not presented in the paper because of their large size and low information value. We have also found this phenomenon in the other salmonids (Atlantic and Far-Eastern salmons, trout, and chars), which is probably associated with their polyploidy origin. We used sperling (*Osmerus *sp.) as an outer group; in our opinion, notably lower degree of DNA banding patterns in this species could testify in behalf of this assumption.

**Figure 2:**
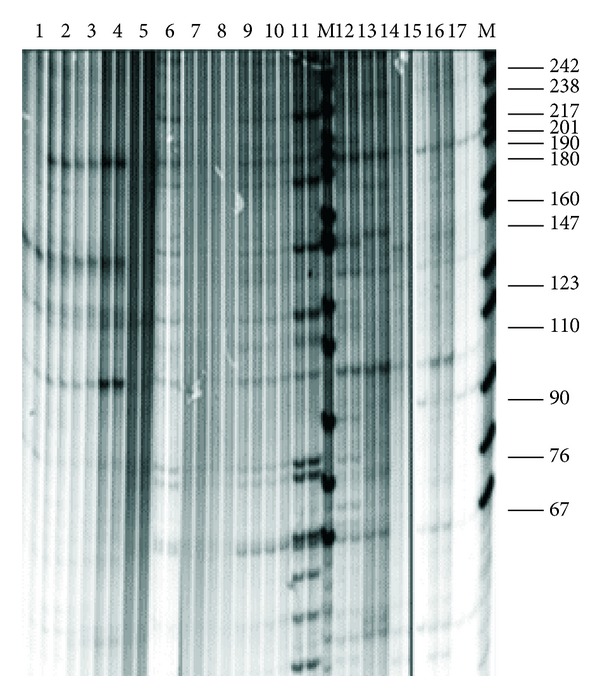
Electrophoretic separation of DNA *Taq*I digest from (1) *Salmo salar*; (2) *S. trutta*; (3) *S. trutta caspius*; (4) S. *ischchan*; (5) *Parasalmo mykiss* (steelhead, anadromous form); (6) *P. mykiss* (with traits of *P. clarkii*); (7) *P*. *mykiss* (freshwater steelhead); (8) *P. mykiss* (introduced rainbow trout); (9) *P*. *mykiss* (steelhead, North America); (10) *P. clarkii*; (11). *Oncorhynchus masou*; (12) *O. keta*; (13) *O. gorbuscha*; (14) *O. nerka*; (15) *O. kisutch*; (16) *O. tshawytscha*. M-marker: *Msp*I-digested pBR322 DNA.

**Figure 3:**
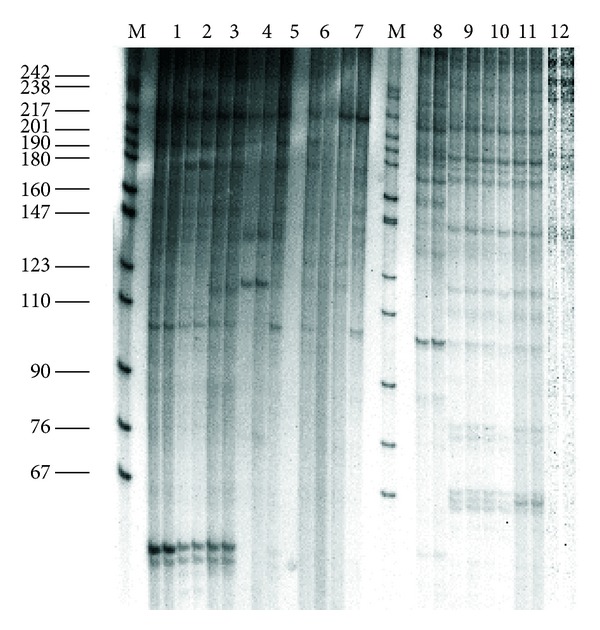
Electrophoretic separation of DNA *Taq*I digests from: (1) *Salvelinus alpinus*; (2) *S. drjagini*; (3) *S. malma*; (4) *Salvethymus svetovidovi*; (5) *Salvelinus elgyticus*; (6) *S. boganidae*; (7) *S. confluentus*; (8) *S. leucomaenis*; (9) *Parasalmo clarkii*; (10) *P. mykiss*; (11) *Salmo irideus*; (12) *Oncorhynchus masou*. M—marker: *Msp*I-digested pBR322 DNA.

**Figure 4:**
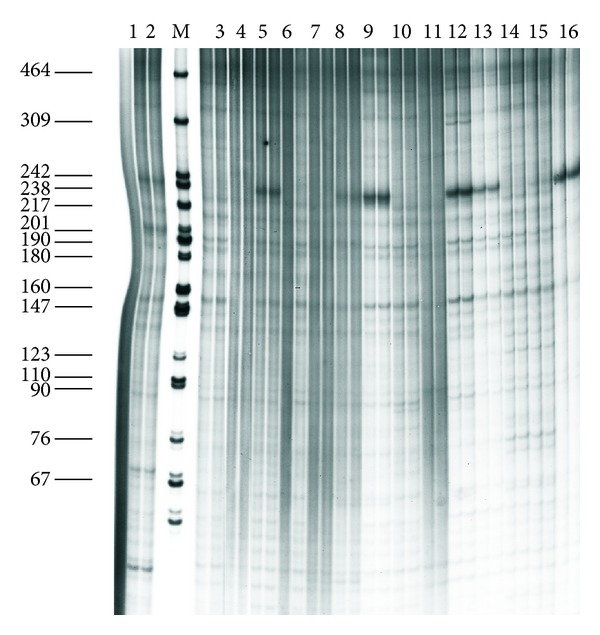
Electrophoretic separation of DNA *Taq*1 digests from (1) *Osmerus* sp. (outgroup); (2) *Coregonus lavaretus* lacustrine form; (3) *C. lavaretus* anadromous form; (4) *C. autumnalis migratorius*; (5) *C. nasus*; (6) *C. lavaretus montchegor*; (7) C. *muksun*; (8) *C. lavaretus pidschiani*; (9) *C. albula*; (10) *C. sardinella*; (11) *Stenodus leucichthys nelma*; (12) *Prosopium cylindraceum*; (13) *C. autumnalis* (Como Lake); (14) *C. clupeaformis* (Como Lake); (15) *C. nasus* (Como Lake); (16) *C. artedi* (Huron Lake).

The second characteristic trait is the high degree of similarity of the repetitive DNA in the studied fishes, which not only are “good” morphological species but also belong to different genera. On the basis of this characteristic whitefishes differed from all previously studied organisms [25], the fishes of Salmonidae family (genera *Salmo*, *Parasalmo*, *Oncorhynchus*, and *Salvelinus*—Figures 2 and 3) among them; see also [27] and [45]. An example of whitefishes' restriction pattern is shown on Figure 4.

The results obtained indicate that each whitefish species is characterized, under chosen experimental conditions, by a number of major and minor bands ranging between 600 and 40 bp, but only some of them are species specific. For example, major band of 230 bp appearing after digestion by *Taq*I restriction endonuclease is present in broad whitefish *Coregonus nasus*, the least cisco *C. sardinella,* and three North American species: Arctic cisco *C. autumnalis*, broad whitefish *C. nasus*, and lake cisco *C. artedi*. This band is notably less pronounced in *Msp*I cutting *C. lavaretus* from Lake Anetti, Finland (predator, high-gillraker form) and European cisco *C. albula*. The rest of species under study lack this band. The band of 150 bp is common to all whitefish species except for round whitefish *Prosopium cylindraceum*. Specific bands correspond to 300 bp in Canadian Arctic cisco and 80 and 60 bp in Finnish high-gillraker whitefish. Distinct species-specific band of 170 bp appears in round whitefish after digestion by *Msp*I; the rest major bands of 460 bp, 310 bp, and 90 bp and smaller are common for all studied DNA. The sites for *Sau3A*I and *Tru9*I restriction endonuclease are not polymorphic at all. *Cfr13*I (*Asu*I) reveals species-specific bands 180 bp and 190 bp in round whitefish; the rest major bands (350 bp, double 300 bp, 240 bp, 150 bp, 135 bp, double 100–110 bp and smaller) are identical in all studied DNA. 

Thus, taxonoprint analysis revealed surprising homogeneity of the fraction of high-copy DNA repeats in different species and even genera of whitefish (with round whitefish being the single exception mentioned above). Most of the major bands in the patterns of all used restriction endonucleases were absolutely identical. Slightly pronounced polymorphism was found only in the families of sequences with small number of copies. Reliability of the relative positions of the branches on computer-generated phylogenetic trees appeared to be low.

Taxonoprint analysis of *Salmo*, *Parasalmo,* and *Oncorhynchus* genera specimens (Figure 2) showed distinct division of the samples under study into four groups of generic rank: (1) Atlantic salmon *Salmo salar*; (2) Brown trout *Salmo trutta*, Caspian trout *Salmo trutta caspius*, and closely related trout *Salmo ischan* from Lake Sevan; (3) Kamchatka rainbow trout *Parasalmo mykiss*, its American freshwater form *P. gairdneri*, and cutthroat trout *P. clarkii*; (4) other species of the Pacific salmons *Oncorhynchus* genus. The bands of 242 and 240 bp, 175 and 140, 120, 110, and a number of bands lower than 70 bp revealed by the use of *Taq*I (Figure 2) are genus specific for all *Salmo sensu stricto* species. The bands of 240 and 150, 120 and 76, and short bands of 10, 20, and 30 bp absent in Atlantic *Salmo* and in *Oncorhynchys sensu stricto* [27] were present in the American and Kamchatka trout. The number of species-specific bands appeared to be very small, with coho salmon *Oncorhynchus kisutch* being the sole exception. However, the DNA pattern of masu *Oncorhynchus masou* has nothing in common with the patterns of Pacific trout and Atlantic salmons, except for the family-specific bands of 67 bp, 110 bp, and 510 bp (Figure 2); the rest *Oncorhynchus* species differ from them to even greater degree. 

**Figure 5:**
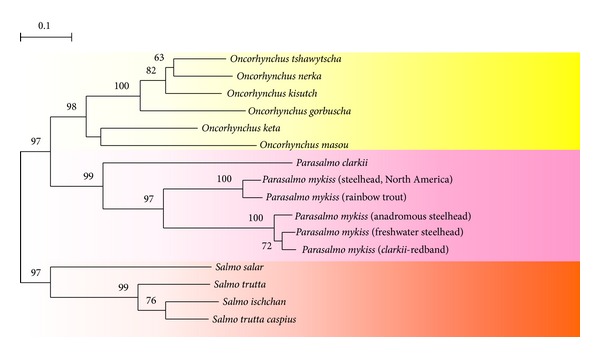
Salmon fishes NJ unrooted tree from restriction analysis data.

NJ dendrogram constructed on the basis of taxonoprints of repetitive DNA of salmonid fishes of *Salmo*, *Parasalmo,* and *Oncorhynchus* genera is shown in Figure 5. All six species of the *Oncorhynchus* genus in its classical interpretation form, with high reliability, a cluster isolated from the American and Kamchatka trouts. 

Distance analyses of endonuclease restriction data and trees reconstruction were done with UPGMA [43]. Figure 5 depicts the genetic variability within salmon (a), chars (b), and whitefishes (c).

Dendrogram (a) of the Salmonidae family represents a robust phylogeny of species with perfect reproductive isolation. Although position of some branches is controversial, the divergence of the Pacific trout *Parasalmo* and the salmon *Oncorhynchus* is firmly established. The diversification of major nodes is dated by 5–15 Mya [46, 47]. 

Within the genus *Salvelinus* (b), all debatable species of the *Salvelinus alpinus* − *Salvelinus malma* complex (including the North American Dolly Varden *S. confluentus*) are separated by small genetic distances. Their phylogenetic relationships cannot be established, as evident from low bootstrap support values. The compact *Salvelinus alpinus* − *Salvelinus malma* complex separates from *Salvethymus svetovidovi* and the “good” species *S. leucomaenis*, although the genus rank of *Salvethymus* [45, 48] cannot be confirmed with these data. The genus *Salvelinus* diverged at least 10 Mya [49], although the *Salvelinus alpinus* − *Salvelinus malma* complex is of glacial or postglacial origin, that is, of age less than 1 My. 

On the whitefish phylogeny (c) the unresolved node contains all European and Asian species and forms of *Coregonus*. The North American representatives of the genus form a robust monophyletic clade. However this group is also compact, and the distance between the two contained taxa is less than 0.2 scale units. The basal branch leads to the second coregonid genus represented by *Prosopium cylindraceum*. Recent whitefish diverged in the Pliocene 2–5 Mya or, perhaps, even earlier [50]. The genus *Prosopium* diverged in the early Miocene [51] dating the origin of round fishes to 20 Mya. This is the only reproductively isolated species of the Coregonidae highly supported as a distinct lineage by repetitive DNA restriction data. 

**Figure 6:**
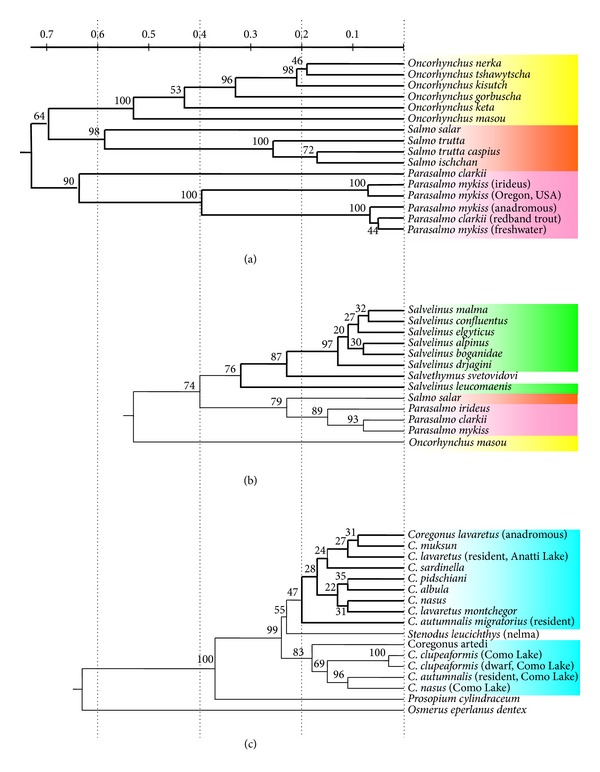
The extent of genetic variability within salmon observed from restriction endonuclease digestion data by UPGMA. (a) Salmonidae, (b) *Salvelinus*, and (c) Coregonidae. For (a) and (c) the proportion of acrylamide/bisacrylamide in PAGE was 29 : 1; for (b) this proportion was 19 : 1, which resulted in slightly lower numbers of detected bands and lower absolute genetic distances.

Genetic variability of highly repetitive DNA in the three groups (Figures 6(a)–6(c)) is in good agreement with dating of the clades and the level of reproductive isolation: high within true salmons (clades diverged about 15 Mya; species are strictly isolated), intermediate within chars (the genus itself diverged about 10 Mya, the *Salvelinus alpinus* − *Salvelinus malma* complex—less than 1 Mya; forms are genetically close, albeit their hybridization is spatially prevented), and low within whitefishes (the main diversity established 2–5 Mya; hybridization is commonly observed across all Eurasian forms).

The presented evidence experimentally corroborates the hypothesis of concerted evolution of long satellite DNA.

### 3.2. RAPD-PCR

**Figure 7:**
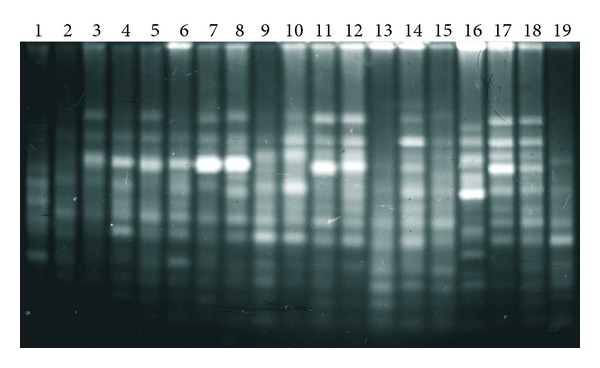
PCR-RAPD electrophoretic pattern of coregonid fishes DNA with pairwise combination of primers no. II + IV (Table 2). (1) *Osmerus *sp.; (2) *Coregonus lavaretus* (resident high-gillraker form, Anatty Lake); (3) *C. lavaretus* (anadromous form); (4) *C. lavaretus* (resident form); (5) *Coregonus lavaretus montchegor*; (6) *C. lavaretus pidschiani*; (7) *C. muksun*; (8) *C. autumnalis migratorius* (Baykal Lake, resident form); (9) *C. autumnalis autumnalis*; (10) *C. autumnalis* (Como Lake, resident form); (11) *C. nasus*; (12) *C. nasus* (Como Lake); (13) *C. albula*; (14) *C. sardinella*; (15)* Stenodus leucichthys*; (16) *Prosopium cylindracium*; (17) *C. clupeaformis* (Como Lake); (18) *C. clupeaformis* (dwarf); (19) *C. arthedi. *

**Figure 8:**
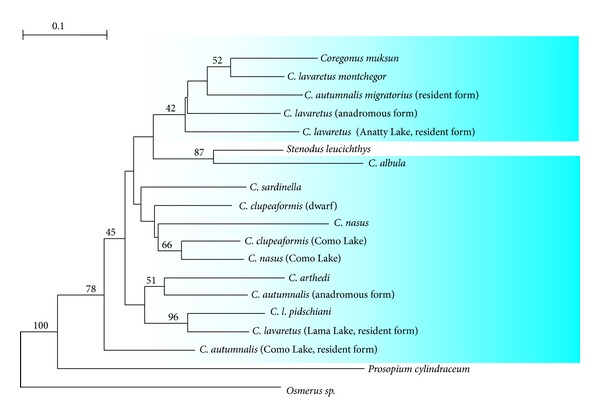
NJ tree of coregonid fishes from PCR-RAPD (more detailed description of species and forms are at the legend of Figure 6).

As could be expected, data on PCR-RAPD of whitefish were more diversified (Figure 7), though in this case bootstrap indices also appeared to be not high when the phylogenetic trees of Coregonidae were constructed (Figure 8). 

Changes in algorithms of calculation of the genetic distances resulted in alteration of the tree topology. With all obtained dendrograms being considered, we can probably discuss only certain tendencies in relationships of whitefish. Round whitefish *P. cylindraceum* stands apart from all other species in the family Coregonidae and tends to occupy the position of the outer group. It shows up in all trees constructed by the UPGMA method when the outer group is chosen automatically as the most distant one. That is to say, remoteness of round whitefish *P. cylindraceum* from the other studied whitefishes is comparable to that of the sperling *Osmerus *sp. Inconnu (nelma) *Stenodus leucichthys* forms cluster with cisco *Coregonus albula*, whereas Baikal omul-arctic cisco (*C. autumnalis migratorius*) should be separated from the Arctic cisco *C. a. autumnalis* as an independent species. The latter is very close to the Bering cisco *C. laurettae* and has probably originated from the *Clupeaformis arthedi* group of numerous whitefish species inhabiting the Great Lakes. This assumption matches data on allozyme analysis [52]. The Baikal Arctic cisco *C. autumnalis* have been already proposed to be segregated as a separate species [53]. Our data suggest that *Leucichthys* is a composed diphyletic subgenus. 

**Figure 9:**
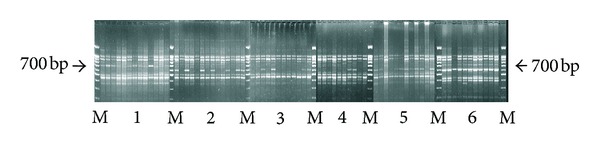
PCR-RAPD electrophoretic pattern of chars genus *Salvelinus* DNA with pairwise combination of primers II + 3. The samples are (1) Dolly Varden—*Salvelinus malma* (Kol' River, Kamchatka Peninsula); (2) Dolly Varden (Chernoe Lake, Onekotan Island); (3) Dolly Varden (Fontanka Stream, Onekotan Island); (4) Dolly Varden (Shelekhovka River, Paramushir Island); (5) Dolly Varden (Gol'tsovyi Stream, Onekotan Island); and (6) white-spotted charr—*S. leucomaenis* (South Kuril Islands). M—marker ladder 100 bp + 1.5 kb.

Experiments on RAPD analysis of *Salvelinus* chars were aimed at the assessment of the genetic diversity of the wild populations of *Salvelinus malma* inhabiting Paramushir and Onekotan Islands. Electrophoretic pattern is shown in Figure 9.

Population genetics of *Salvelinus* is of particular interest because of extensive processes of form and species generation occurring now in the genus [54]. Nominal species of the genus are polymorphic and represented by anadromous and freshwater forms as well as the geographic isolates characterized by particular morphological traits. Despite great commercial interest in salmonid fishes, the rate of genetic isolation of various char forms in the wild has been poorly studied so far. DNA of five available “good” morphological species (*S. alpinus, S. leucomaenis, S. fontinalis, S. namaycush, and S. malma*) was used for selection of the primers that allow discrimination between the Salvelinus species. The layout of electrophoretic patterns of the reactions with the pairs of primers NN 1, 9, 11, 12, 15, 30, 31, 32, 35, 37, 58, 69, and 76 (Tables 1 and 2) was used. The listed markers had sufficient polymorphism and the difference between the species is evident from the pattern of the major bands as well. In the course of binary matrix compilation, all bands in the electrophoretic pattern of PCR products were taken into consideration. 

A total of 170 DNA samples representing 23 forms and species of chars were analyzed for the tree construction. Many analyzed isolated forms have been described [55] as species on the basis of various phenotypic characteristics, though some experts [54, 56] consider them to represent a single complex species *S. alpinus *complex with circumpolar distribution and low rate of genetic distinction [57, 58]. On this particular stage of the study we attempted to use genetic distances in a restricted system of the markers as a measure of taxonomic status of the certain forms. *Salmo salar* was used as an outer group. We took advantage of the matrix of pairwise distances, generated by TREECON for NJ tree construction to comprise the absolute values of genetic distances between commonly acknowledged “good” as well as disputable species and isolates (Table 3). On the tree as well as in the table the disputable species are designate by quotation marks.

**Figure 10:**
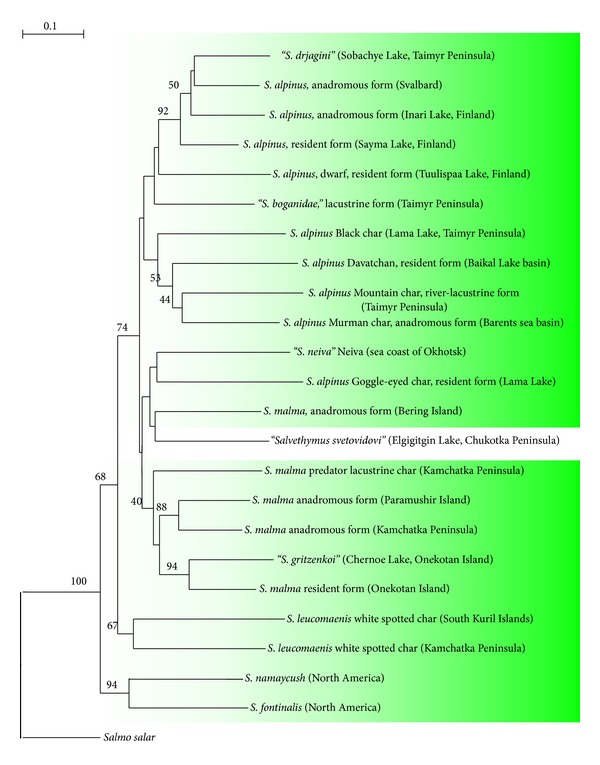
NJ tree of the g. *Salvelinus* forms and species, constructed in accordance with Nei genetic distances. The figures at the node indicate bootstrap indexes that exceed 40%.

Analysis of the genetic distance dendrogram (Figure 10) allows us to draw the following conclusions. The tree consists of three clusters. The first of them brings together all forms and species of *Salvelinus alpinus *complex under study. The stability of this node is 74% of bootstrap. The entire assembly of Dolly Varden (*S. malma*) is included into this cluster as its component. It is heterogeneous in the used system of the markers. Anadromous Dolly Varden from Bering Island appeared to be proximal to the long-finned char *Salvethymus svetovidovi* from Lake Elgygytgyn, though this grouping is uncertain. On the whole, the entire node combining the malma trout and the forms of the Arctic char seems unsolved. Kamchatka predatory char (*S. malma*) forms complex with the other Kamchatka and Kuril Dolly Varden (40% bootstrap support). UPGMA method even more robustly adds predatory char to the cluster containing Dolly Varden (tree is not presented). The same common cluster, along with the *S. malma*, contains also long-finned char “*Salvethymus*,” described as a specimen of a separate genus [46].

Since at this stage of the work resolving power adequate to the species or close to it taxonomic level was used as a criterion for selection of the markers, 46% support for the inner cluster suggests the Dolly Varden being, more likely, of intraspecific status with respect to *S. alpinus* complex, as Berg supposed [59]. The second supported cluster (67% of bootstrap) is formed by white-spotted char *S. leucomaenis*. Reliability of the specific status is undisputed in this case. Although genetic distance between the South Kuril and Kamchatka white-spotted chars is relatively high, they form a monophyletic tree cluster corresponding to the single species *S. leucomaenis*. At least, two American species, *Salvelinus fontinalis* and *Salvelinus namaycush,* form the third branch supported by 68%. Alteration of the outer group (*Salmo salar* changed for *Osmerus*) or application of the alternative methods to construction of the trees (UPGMA or parsimony) did not change the topology in the basal part. In fact, two North American species represented an additional outer group for all other members of genus *Salvelinus*.

Within three discriminated clusters, the branch points are in most cases uncertain and could hardly be used for determination of the relationships of the separate populations. The dendrogram shows that anadromous Arctic char *Salvelinus alpinus*, a type species of the genus *Salvelinus*, forms a compact group that includes both anadromous and freshwater forms from Inary and Sayma Lakes (Finland), anadromous char from Svalbard, and Drjagin's char from Taimyr. According to Table 3, intrasample distances in this group range between 11.4 and 23.5 Nei's distance units (×10^−2^) and averaged 18.03 Nei's units. In parsimonial construction the cluster shows maximal number of synapomorphies (not shown).

This group was used as a reference point for the estimation of the average genetic distances between chars of different taxonomic status. The distances between the samples of Dolly Varden (24.8 × 10^−2^ Nei's units on average) were estimated likewise. Subsequent analysis of the correspondence of the genotypic data to the position of particular forms in the system of genus *Salvelinus* was performed with the use of the table of pairwise distances. Data presented in Table 3 allows comparison of the absolute genetic distances between various forms of chars with the disputable taxonomic status and the “good” species: arctic char *Salvelinus alpinus*, white-spotted char *S. leucomaenis*, brook trout *S. fontinalis*, and lake trout *S. namaycush*. According to the table, the distances between *S. alpinus* and *S. leucomaenis*, and *S. fontinalis* and *S. namaycush* exceed 40 × 10^−2^ Nei's units. The distance between all the rest chars and *S. alpinus* could be defined as corresponding to the intraspecific taxonomic level with the different rate of advance. The longest distances are characteristic of the isolated forms of chars inhabiting the river and lake basin of the Taimyr Peninsula and Transbaikalian endemic species [60]. Although the data of this table are by no means the absolute indicators of the taxonomic status of particular forms, they provide an idea about correspondence of the phenotypic and genotypic data. 

Identification of the nucleotide sequences of the fragments generated in PCR in the used system of primers with subsequent search for the homologous sequences in GenBank showed that most of the fragments forming RAPD-PCR electrophoretic pattern had homologies in dbEST base of NCBI resources with the “Salmonidae” or “zebrafish” as a filters (Table 4). Only extensive homological sequences with low “expect” value (low probability of the random coincidence) were inserted into the table.

RAPD fragments for the sequencing were chosen from North Kuril Islands populations *Salvelinus malma* DNA. From 160 clones analyzed for 10 insert DNA the homologies were not found (“junk” DNA?) and for four inserts homologies were found in nucleotide collection (microsatellites). The great part of the salmon fishes EST resources where homologies for variable RAPD fragments have been found was developed by Koop et al. [61]. The detailed analysis of these results is still not completed now, but major pattern appears to be followed. It was found that the majority markers used were either entire exons or contained exon fragments, abundantly exhibited in cDNA libraries, and portions of introns or intergenic DNA without detectable homologies in any library. The sequences with open frame encoded conservative protein domains in individual cases were found for the molecular markers with exon origin. For example, a significant and extensive homology with the hypothetical variable protein of *D. rerio* with the length of 659 amino acids (acc. no. XP 001332830.1), 52% identity, and very low probability of coincidence (2^−26^) was found after the nucleotide matrix of 12.3 fragment had been converted into translated protein sequence by reading from frame 3. 

Another fragment with the length of 905 bp was found to be the most homologous to *D. rerio *(zebrafish): clone DKEY-182H7 in linkage group 7 (acc. no. BX 663609.29) and mRNA of predicted protein, similar to norepinephrine (analogue of noradrenalin) carrier (acc. no. XM 689046.3 in GenBank). All the remaining homologies found for this sequence were to certain extent connected with noradrenalin protein carriers.

As was pointed out above, all PCR-RAPD reactions were repeated two times as minimum and reproduced absolutely. May be it is the result of the RAPD markers connections with the conservative protein coding genome areas in used primers system. We analyzed the monomorphous, generating the structure of electrophoretic patterns as well as polymorphic fragments. Essentially, it seems likely that introns or repetitive “junk” DNA with no homologies in dbEST or nucleotide databases are most informative for estimating genetic distances. For some of them the homologies in NCBI dbTSA were found. 

Only in the single cases the homological sequences belonged to mtDNA with high evolution rate that should be able to correspond with the divergence in intraspecific taxonomical level. For example for reading from frame 4 sequence of 254 bp length (Table 4) the homology was found with Cytb of Atlantic salmon *Salmo trutta* (acc. no. ACO57211). However, it is impossible to exclude the implication of adaptive nuclear sequences in divergence of model groups studied.

Since the fish's groups selected differ considerably in basic stages of evolution and divergence time [30, 47, 51, 62, 63], integrated genomes assessment allows reducing the errors of disparity caused by difference in molecular evolution of different genes [64].

In the row containing almost all species and isolated forms of chars (disputable allopatric “species”), absolute genetic distances were used as a criterion—see Thorpe [65]. Correctness of this approach was confirmed by 100% homology of nucleotide sequences of RAPD fragments with identical electrophoretic mobility obtained in a single reaction. 

According of our results the well-identified species, the lake trout and the brook trout, form a single cluster. No information on the level of phylogenetic relationships between *S. namaycush* and *S. fontinalis* is available to us. However, existence of hybrid forms traced up to F_4_ by Berst et al. [66] and entering reproductive relations indicate close relations between these two North-American species. Molecular data by Westrich et al. [67] also are in favor of this assumption. Sister relations between *S. namaycush* and *S. fontinalis* oppose the proposal to consider *S. namaycush* as a specific genus “*Cristivomer*” [68]. This species should be placed in genus *Salvelinus*. 

The second conclusion is connected with extremely low values of genetic distances between all samples of Dolly Varden and forms of the Arctic char. The degree of these distances is the same as that between the anadromous and isolated forms of the Arctic char, and even lower in some cases. If we rely on the distances only, we should refer all studied populations of Dolly Varden to the Arctic char. In other words, specific status of *Salvelinus malma* could be contested in case it is based only on the absolute values of genetic distances. 

Restriction analysis of the Atlantic and Pacific salmons has revealed specific sets of DNA fragments in every species and even in the morphological forms of these fishes. Along with it, the results indicate pronounced isolation of the Pacific trout from the members of the genus *Oncorhynchus*. In addition, taxonoprints considerably and reliably differ in the species with overlapping ranges (sympatric species). It is true for Pacific salmons of the genus *Oncorhynchus* (chum salmon, pink salmon, sockeye salmon, chinook salmon, cherry salmon, and coho salmon), the chars of the genus *Salvelinus* (*S. alpinus* and *S. leucomaenis*), and two chars from Lake Elgygytgyn (long-finned char *Salvethymus svetovidovi* and Arctic char *S. alpinus*). 

The main part of these results was received with anonymous DNA sequences before the GenBank recourses became available, but they do not seem contrary to Crespi and Fulton [69] strong results with employment of a powerful tool of genomics (with the exception of taxonomic relations of *O. masou*).

Analysis of whitefishes' restriction data yielded the results not completely matching those generally accepted in the modern systematics of whitefishes [50, 70, 71]. From the point of view of systematics, Coregonidae is one of the most complex and intricate groups. Great variability and polymorphism of the whitefishes are the reason for the differences in conclusions about phylogenetic links between species based on different approaches, for example, Bernatchez et al. [72]; Smith and Todd [73]; Bodaly et al. [52]; Frolov [74], Turgeon and Bernatchez [30]. Investigation of genetic structure of the species and identification of closely related species from various sites of the range also cause difficulties. Up to a hundred intraspecific categories were described for a whitefish type species *C. lavaretus* from the Russian water basins only [50]. 

Interspecific and even, in some cases, intergeneric hybridization between the representatives of Coregonidae family yielding viable hybrids is a well-known fact, described by Garside and Christie a long time ago [4]. Casual relations between the diversity of the forms of whitefishes and introgression have been discussed more than once, for example, Svärdson [75] and Bernatchez et al. [76]. The possibility of exchange of genetic information in whitefish could be considered proved. It could be due to this that the index of genetic distances in them is usually lower than that in the taxa of the same level in other animals as reported by Bodaly et al. [52] and Kartavtsev [77]. Such phenomenon as “genetic parasitism,” when the smaller and more numerous form replaces the much larger one, has been found and documented by Svardson in whitefishes [78]. Geographic isolation and isolation of other kinds as well as relatively young historical age (the main diversification of *Coregonus* species dates to Pleistocene glaciations—about 15,000 years in accordance with Behnke [51]) must have prevented various Coregonidae species from developing specific families of repetitive sequences, as it is common to noninterbreeding populations. On the contrary, many families of repetitive sequences are homogenous within all these forms, which indicate the intensive gene flow between all these species allowing molecular drive to adjust them. 

All above mentioned enlightens us about greater rate of the differences in the experiments on amplification with arbitrary primers. They are the markers of the loci of expressed sequences and introns that are not subjected to molecular drive. However, no differences sufficient for reliable discrimination of the species have been accumulated in these fractions either. Taking all the aforesaid into consideration, we can state that all these facts summed up vividly indicate a peculiar way of evolution and genetic structure of the Coregonidae population. Contrary to the common divergent evolution characteristic of the majority of animals, whitefishes demonstrate the elements reminding of reticulate evolution (“evolution via hybridization”), as supposed by Todd and Smith [29], Turgeon, Bernatchez [30], and Svardson [79], described for many plants, for example, Grant [80]. This pattern of evolution implies alternation of the divergent and hybridization (conversion) stages. External phenotypical differences in this case are determined by a small number of genes acting as morphological triggers and switching morphogenesis to a certain direction; the final studies of the process are known as discrete described forms written by Renaut and Bernatchez [81]. Accumulated cross breeding of the hybrids with prevailing parental species could become one of the possible mechanisms supporting morphological independence in the course of interspecies exchange of genetic information. However, any interpretation based on genetic isolation of Coregonidae species in the wild encounters the following unsolvable inconsistency. In the framework of such traditional hypothesis, one had to explain why the mutations in DNA repetitive sequences no longer occurred and were not preserved in this group, which seems incredible. Thus, the results obtained with the use of restriction analysis of highly repetitious DNA revealed great differences between electrophoretic patterns of the salmons with the high degree of reproductive isolation and the whitefishes. In the latter, introgressive hybridization has probably occurred, and the species still hybridize retaining their independent status.

Geographic isolation of the species is one of the mechanisms preventing interspecific mating. Over a long time, the view on allopatric speciation as a process of gradual accumulation of gene mutations of adaptive character located mostly in the coding DNA sequences has been commonly accepted (see, e.g., [82, 83]), and noncoding repetitive sequences were thought to be “junk” DNA. Now indirect evidence of involvement of the repetitive sequences (mostly mobile elements) in the adaptive evolution has been suggested by many researches—see for review Schmidt and Anderson [84] and Osada and Wu [83]. Studies of two sister *Drosophila* species have shown that heterochromatic region of X chromosome plays a great role in the establishment of the reproductive barrier [85]. Our results support this viewpoint.

## 4. Conclusion

The families of salmon fishes with different levels of reproductive isolation were compared using two strategies of multilocus fragment analysis. The compared lineages are (1) Salmonidae, possessing almost perfect homing and absolute reproductive isolation; (2) chars of g. *Salvelinus* (f. Salmonidae), possessing “good” and “difficult” species (reproductive isolation is often of spatial nature); (3) g. *Coregonus* (f. Coregonidae), containing recognized taxonomic species with common interspecies hybridization but distinct species phenotypes. Each sampling contained intraspecies forms and/or “disputable” species, “good” species, interbreeding species, and representatives of a sister genus. 

Genetic distances were compared for lineages of the same taxonomic rank and juxtaposed with their divergence times; bootstrap support values were verified for corresponding phylogenetic clades. As a markers two types of sequences were chosen: (1) the long regions of satellite DNA, and (2) anonymous loci containing in 70% cases conserved exon and intron (or intergenic) regions. Genetic distances and clades robustness were shown to correlate well with the level of reproductive isolation in both marker systems. 

The hypothesis of concerted evolution of satellite DNA is experimentally corroborated. The stronger is reproductive isolation between forms and species; the more species-specific band patterns are found in satellite DNA. Among whitefishes, the round fish *Prosopium cylindraceum* is the only reliably distinguished species separated from the unresolved *Coregonus* clade by a genetic distance comparable to those between individual genera in Salmonidae.

Molecular markers were used to clarify particular questions in salmon taxonomy and systematics: (1) *Salvelinus fontinalis* and *Salvelinus namaycush* are genetically close to each other; (2) *Salvethymus svetovidovi* cannot be considered a separate genus; (3) Dolly Varden *Salvelinus malma* is genetically identical to *Salvelinus alpinus*; (4) all species of the genera *Salmo*, *Parasalmo,* and *Oncorhynchus* are reliably distinguished, with the genus *Parasalmo* being sister to *Oncorhynchus*.

## Figures and Tables

**Table 1:** The RAPD-PCR primers sequences.

No.	Designation	Sequence 5′-3′	Length [nucleotides]
1	I	CGT TGG AAG ACA GAC CTC CG	20
2	II	ATT CCC TGT CAA AGT AGG GT	20
3	III	GAG CAC TTT CTT GCC ATG AG	20
4	IV	GAA GCT GCT ATG CTT CGT AT	20
5	VI	CAT AAA TTG CTT TAA GGC G	19
6	VII	TCA TCT TCT TCC TCT TCT TC	20
7	1	TGT GAC TGC TTG TAG ATG GC	20
8	2	TGG AGC TGT GTA AGA AGT AC	20
9	3	AAA AGA CAT GAA GAC TCA GG	20
10	5	TGG ACA GTA CGG TGA ATG C	19
11	6	CCA CAA ACC AAT ATC TCT C	19
12	7	CTC AGA GTC CAA CCT GGG TAG	21

**Table 2:** Combinations of oligonucleotides, used in pairwise RAPD-PCR.

Pair no.	Combination
1slv	1 + I*
4sm	1 + IV
9slv	2 + II
11slv	2 + IV*
12slv	2 + V
14sm	2 + VII
15slv	3 + I*
16sm	3 + II
18sm	3 + IV
30sm	5 + II**
31sm	5 + III**
32sm	5 + IV**
32′sm	5
35slv	5 + VII*
37sm	6 + II**
43sm	7 + I
44sm	7 + II
45sm	7 + III
56sm	2 + 3
58slv	2 + 5*
69sm	5 + 7*
71sm	I + II
76slv	I + VII*
77sm	II + III

(*) Pairs, used for the genus *Salvelinus *species and forms (**)—Pairs, used with all the rest fishes DNA.

**Table 3:** The absolute values of genetic distances Nei between different forms of chars and the valid species.

Species and forms	S. alpinus*	S. leucomaenis*	*S*. *fontinalis *	*S. namaycush *
Genetic distances [Nei × 10^−2^]				

*S*. alpinus*	0.0	40.35	43.0	41.6
*S*. *alpinus* (Tuulispaa Lake)	25.8	41.6	48.1	42.1
*S*. *alpinus* Black char (Lama Lake)	34.6	44.4	53.3	50.0
“*S*. *boganidae*”	27.55	38.35	40.3	40.4
*S*. *alpinus *(Barents Sea basin)	27.1	44.55	50.0	46.1
*S*. *alpinus* Davatchan (Baikal Lake basin)	33.4	46.35	56.5	53.3
*S*. *alpinus* Mountain char (Lama Lake basin)	34.17	44.4	47.2	49.6
*S*. *malma* Predator (Kamchatka Peninsula)	27.85	40.1	41.9	36.4
*S*. *alpinus* Goggle-eyed (Lama Lake basin)	34.7	4.9	46.4	48.8
“*S*. *neiva*” Neiva (Sea of Okhotsk basin)	30.3	45.3	40.5	48.3
“*Salvethymus svetovidovi*” (Elgigitgin Lake, Chukotka Peninsula)	26.7	46.6	41.5	40.2
*S*. malma* (Kuril Islands)	29.26	40.6	40.3	41.9

The matrix of distances was generated by TREECON for construction of NJ tree. For the chars that abbreviate with (*) the averaged pairwise distances between all samples analyzed are shown. The more detailed description of sample is shown at Figure 10.

**Table 4:** *Salvelinus malma* DNA PCR-RAPD fragment sequences searched for NCBI.

Pairs of primers	PCR RAPD cloning fragment length**	Genbank Accession Numbers of homological sequences*		
		Microsatellites 5′→3′	dbEST*	Protein product***
IV + IV	555 bp		EG849746	
1 + IV	568 bp		DY73125	
IV + IV	463 bp		EG827041	BT_071991
1 + 1	304 bp		EG827041	
IV + 1	439 bp		EG930580	NP_001133464
IV + 1	676 bp		BX086452	
IV + IV	446 bp		CX357234	
IV + 1	395 bp		EG935616, DW006459	XP_003198377
1 + 1	418 bp	Microsatellite (CA)n 173–331		
1 + 1	905 bp		BX663609	XM 689046
2 + VII	409 bp	Microsatellite (GT)n 5–32		
3 + 3	549 bp		EG923517	NM_001102593
3 + 3	384 bp		DQ156149	XP_001332830
3 + 3	384 bp		CB511135.	
II + 3	122 bp		EU621899	XP_001336520
II + II	959 bp	Microsatellite (GT)n 226–294	AU081124	
3 + II	283 bp		CX723014	
3 + II	453 bp		CU062733	
3 + II	190 bp		GE828193	
3 + II	130 bp		GU552297	ADV31329
IV + 3	108 bp		EG911815	XP_003385009
3 + IV	236 bp		EG911136	ACH85273
3 + IV	389 bp.	Microsatellite (CA)n 276–329		
3 + IV	413 bp		EG792115	CBX11156.
3 + IV	724 bp		CA353611	
IV + 3	109 bp		EG911815	XP_001195378
3 + 3	801 bp		DW556963	ACI33792
3 + 3	496 bp		CB486060	
3 + 3	412 bp		EG831541	NP_001154053
3 + 3	322 bp		BX861631	XP_003197666
IV + IV	554 bp		EG849746	NP_001167305
3 + IV	290 bp		EG915402	NP_001135251
3 + 3	638 bp		CB509929	ACO08436
3 + IV	275 bp		CA388004	NP_001133389
3 + 3	685 bp		CK898369	NP_001167187
3 + 3	414 bp		FF839690	
IV + IV	254 bp		CB490887	ACO 57211 AAP58348
3 + IV	236 bp		EG911136	ACI66028
3 + 3	414 bp		EG792114	XP_001335224
3 + 3	490 bp		CB486060	ACI66769
7 + I	330 bp		EG 760735	XP_003200023
3 + IV	281 bp		BX861631	NP_001187967

*Only the first homological sequence from different libraries is exhibited.

**The lengths of cloning PCR fragments are given without considering primers.

***Translation performed by EMBOSS transeq (Sequence Translation Sites) of EBI-EMBL.
